# Outcomes of Esophagectomy in a Tertiary Care Center in Pakistan

**DOI:** 10.7759/cureus.87366

**Published:** 2025-07-06

**Authors:** Muhammad Uneeb, Maaz A Yusufi, Hadi M Khan, Murad A Khan, Alishbah Khan, Hania Iqbal

**Affiliations:** 1 General Surgery, Shifa International Hospital Islamabad, Islamabad, PAK; 2 Surgery, University Hospitals Dorset, Poole, GBR

**Keywords:** esophageal cancer, ivor-lewis esophagectomy, oncological outcomes, pakistan, thoracoabdominal esophagectomy, transhiatal esophagectomy

## Abstract

Introduction: Esophageal cancer (EC) is a disease with a poor prognosis. Surgical management, combined with chemoradiotherapy, is the standard management. However, esophagectomy is associated with high morbidity and mortality. There is limited data on esophagectomy and its outcomes in Pakistan. This study aims to address this gap in the literature.

Methodology: A retrospective single-center cohort study was conducted to evaluate patients undergoing esophagectomy for EC during five years, from January 1, 2019, to December 31, 2023. Patients underwent minimally invasive esophagectomy in the form of laparoscopic-assisted Ivor-Lewis esophagectomy, or open surgery, as left thoracoabdominal esophagectomy and transhiatal esophagectomy. Data regarding intraoperative course, postoperative events, complications, and final histopathology reports were evaluated.

Results: A total of 43 patients underwent esophagectomy for EC. The mean age was 62.2 ± 12.1 years, with 31 (72.1%) male patients. Adenocarcinoma was the most common diagnosis, accounting for 27 (62.8%), with squamous cell carcinoma present in the remaining 16 (37.2%) cases. Neoadjuvant therapy was given to 37 (86.0%) patients. Mean operative time was 288 ± 88 minutes. Twenty-six (60.5%) patients had an uneventful postoperative course. Seven (16.3%) patients experienced minor deviations from the routine postoperative course and were classified as grade 1 in the Clavien-Dindo classification. Higher Clavien-Dindo grades were found in 12 (27.9%) cases, indicating greater morbidity. There were no cases of anastomotic leak or chyle leak. There was one reintervention (2.3%) and one mortality (2.3%). There was no association between the development of complications and gender (*P *= 0.245), presence of comorbidities (*P *= 0.224), or the histopathological diagnosis (*P *= 0.555). The majority of cases were T3 tumors, accounting for 21 (48.8%), while the most common grade was grade 2, with 25 (58.1%) cases. Lymph node involvement was absent in 17 (39.5%) patients. The most common final stage was IIIb, found in 19 (44.2%) cases. R0 resection was achieved in 36 (83.7%).

Conclusions: Esophagectomy with reasonable rates of morbidity and sound oncological outcomes is feasible in a developing country like Pakistan. Complications were found to be independent of gender, comorbidities, and the underlying pathology. Larger, prospective, and ideally multicenter studies with a longer follow-up period are needed from this region.

## Introduction

Esophageal cancer (EC) is a disease with a poor prognosis. It is the eighth most common type of cancer, with an annual 604,100 new cases worldwide (in 2020), and is the sixth leading cause of mortality from cancer, accounting for 6.0% of all cancer deaths worldwide [1]. According to the 2019 Global Burden of Disease study, Asian countries accounted for more than 70% of the EC incidence, prevalence, mortality rates, and disability‐adjusted life‐years (DALYs) [2]. Surgical management in the form of resection and lymph node dissection, along with neoadjuvant chemoradiotherapy (CRT), is the standard management for EC [3,4]. However, esophagectomy is associated with high morbidity, depending on several factors, such as the surgical approach and prolonged operative time [3,5]. Neoadjuvant CRT is another source of morbidity; however, it is a necessary component of EC management. The CRT for EC followed by surgery study (CROSS) trial was a multicenter randomized trial, which demonstrated that the overall survival benefit of neoadjuvant CRT persisted for at least 10 years [4]. Different techniques can be employed for esophagectomy, depending on the location of the lesion and the surgeon’s preference. However, the morbidity associated with these procedures is variable, with some complications being more common following certain procedures [6]. Minimally invasive approaches such as laparoscopic and robotic esophagectomy are associated with decreased morbidity as compared to open surgery, with equivalent oncological outcomes [3,7]. However, due to its inherent technical difficulty, minimally invasive surgery demands advanced laparoscopy and thoracoscopy, and is associated with longer operative times [8].

Pakistan faces a considerable proportion of EC in Asia. The 2019 Global Burden of Disease study found that Pakistan had the highest age-standardized incidence rate (ASIR) (7.86 per 100,000) and the highest age-standardized prevalence rate (ASPR) (11.0 per 100,000) among low sociodemographic index (SDI) countries in Asia [2]. Despite this, the uptake of minimally invasive esophagectomy, in particular, has been slow in Pakistan, with a resultant paucity of local data on this topic [9,10]. Our study aims to help bridge this gap. This is a review of the short-term outcomes of esophagectomy for EC, focusing mainly on the associated morbidity and mortality, as well as the pathological outcomes at a single center in Pakistan.

## Materials and methods

A retrospective single-center cohort study was conducted to evaluate patients undergoing esophagectomy for EC over five years, from January 1, 2019, to December 31, 2023. Approval for the study was obtained from the Institutional Review Board and Ethics Committee (IRB and EC) under protocol number IRB-616-24. All consecutive patients who underwent esophagectomy during the study period were evaluated for inclusion. Medical records were utilized for a chart review. Patients with gastric cancer involving the gastroesophageal junction (Siewert-Stein type III) were excluded [11]. Additionally, patients with benign pathologies, such as an esophageal stricture, were also excluded. Following workup, each case was discussed in a multidisciplinary team (MDT) meeting. The decision regarding the type of procedure performed had been made on a case-by-case basis, depending on the patient’s case and the surgeon’s preference. Patients underwent minimally invasive esophagectomy in the form of laparoscopic-assisted Ivor-Lewis esophagectomy (ILE), or open surgery, as left thoracoabdominal esophagectomy (LTE) and transhiatal esophagectomy (THE). Operative procedural details are briefly described below.

Laparoscopic-assisted ILE

Laparoscopic gastric mobilization was performed, preserving the gastroepiploic vessels. The patient was then placed in the left lateral decubitus position, and a right posterolateral thoracotomy was made. The esophagus was mobilized, and the azygos vein was ligated. Following esophageal resection, the proximal margin was sent for frozen section analysis. A gastric conduit was then formed using linear staplers, with sutured reinforcement of the staple line. The esophagogastric anastomosis was either hand-sewn or stapled. A chest drain was placed, and a leak test was conducted at the end of the procedure using a bougie.

LTE

The patient was placed in the right semi-lateral decubitus position, and a left anterolateral thoracotomy was performed. The diaphragm was opened around the gastroesophageal junction, and the stomach was mobilized. High ligation of the left gastric artery was done. The esophagus was mobilized and transected, and the proximal margin was sent for frozen section analysis. The remainder of the procedure was performed similarly to the ILE.

THE

Laparoscopic gastric mobilization was performed, followed by distal esophageal mobilization through the esophageal hiatus. The esophagus was transected, and the gastric conduit was formed with staplers, either laparoscopically or with the help of a limited upper midline laparotomy. Cervical esophageal mobilization was carried out through the left side of the neck, and the esophagus was resected. The proximal margin was sent for frozen section analysis. A retrosternal tunnel was then created using blunt dissection, and the gastric conduit was pulled up through it to the cervical region, where the gastroesophageal anastomosis was created. Intraoperative frozen section was sent in the majority of cases. Pyloric drainage procedures, such as pyloroplasty or pyloromyotomy, were not performed. Postoperatively, all patients were initially managed in either the surgical Intensive Care Unit (ICU) or the High Dependency Unit (HDU) and subsequently transferred to the surgical floor. The first follow-up visit was scheduled one week after discharge from the hospital, with subsequent visits scheduled thereafter, including an oncology review once the definitive histopathology report was issued.

Demographic data and details regarding the intraoperative course, postoperative events, and complications were collected. Routine follow-up was done after patients were discharged from the hospital, with any deviation from the normal course noted up to one month. Final histopathology reports were reviewed for resection status, grade, pathological stage, and lymph node yield. Staging was done according to the eighth edition of the American Joint Committee on Cancer (AJCC) staging manual [12].

Data were analyzed using IBM SPSS Statistics for Windows, Version 25 (Released 2017; IBM Corp., Armonk, NY). Descriptive statistics were computed for continuous and categorical data. Frequencies and percentages were calculated for qualitative variables. Mean and standard deviation were calculated for quantitative variables, with the median in selected cases. The Pearson chi-square test was performed to assess the effect of gender, presence of comorbidities, and underlying pathology on the complication rate. A *P*-value of less than 0.05 was considered statistically significant.

## Results

A total of 78 patients underwent esophagectomy during the study period; however, 23 were excluded due to underlying gastric cancer, 10 due to benign pathology such as stricture or perforation, and two due to untraceable medical records. The final number of cases included in the study was 43.

The mean age was 62.2 ± 12.1 years, with a minimum age of 31 and a maximum age of 84 (Table 1). There were 31 (72.1%) male patients. Adenocarcinoma (AC) was the most common diagnosis, accounting for 27 (62.8%), with squamous cell carcinoma (SCC) present in the remaining 16 (37.2%) cases. Neoadjuvant therapy was given to 37 (86.0%) patients. Three (7.0%) patients had a feeding jejunostomy before surgery, while seven (16.3%) patients had a feeding jejunostomy made in the same surgery. Intraoperative frozen section was done in 35 (81.4%) of cases.

**Table 1:** Patient demographics. N, number of cases; M, mean; SD, standard deviation; BMI, body mass index; ASA, American Society of Anesthesiologists physical status classification system

Variable	*M* ± SD/*N* (%)
Age (years)	62.2 ± 12.1
Male: Female	31 (72.1):12 (27.9)
Height (cm)	164.2 ± 10.2
Weight (kg)	64.7 ± 16.0
BMI (kg/m^2^)	24.0 ± 5.4
Underweight	4 (9.3)
Healthy weight	24 (55.8)
Overweight	8 (18.6)
Class I Obese	6 (14.0)
Class II Obese	1 (2.3)
Hemoglobin (g/dL)	11.7 ± 1.8
Albumin (g/dL)	3.7 ± 0.5
ASA class	
ASA 1	13 (30.2)
ASA 2	22 (51.2)
ASA 3	8 (18.6)
Comorbidities	25 (58.1)
Diabetes mellitus	10 (23.3)
Hypertension	21 (48.8)
Heart disease	7 (16.3)
Smoking status	
Smoker	7 (16.3)
Ex-smoker	13 (30.2)
Non-smoker	23 (53.5)

Perioperative outcomes are shown in Table 2. The mean operative time was 288 ± 88 minutes, ranging from 132 to 528. The American Society of Anesthesiologists (ASA) physical status classification system was applied preoperatively in all cases [13]. A few exceptional cases skewed the mean of certain outcomes, which is why the median is also reported in such instances. One patient had a prolonged hospital stay of 29 days, including a combined 21 days in the intensive care unit (ICU) and the high dependency unit (HDU). This patient was an ex-smoker who had several comorbidities, including diabetes mellitus, hypertension, ischemic heart disease, and atrial fibrillation, as well as a history of stroke. Postoperatively, he underwent placement of a chest drain for a pleural effusion, endoscopic placement of a nasogastric tube, and a tracheostomy for prolonged intubation. Another patient had a rather high volume of blood loss (600 mL), resulting in a higher mean blood loss of 161.9 mL, while the median blood loss was 100.0 mL.

**Table 2:** Perioperative outcomes. N, number of cases; M, mean; SD, standard deviation; ICU, intensive care unit; HDU, high dependency unit

Variable	*N* (%)/*M* ± SD
Type of surgery	43 (100.0)
Laparoscopic-assisted Ivor-Lewis	34 (79.1)
Transhiatal	4 (9.3)
Thoracoabdominal	5 (11.6)
Operative time (minutes)	288 ± 88 (Median: 272)
Blood loss (mL)	161.9 ± 170.0 (Median: 100.0)
Intraoperative transfusion	10 (23.3)
Postoperative transfusion	9 (20.9)
Hand-sewn: Stapled anastomosis	37 (86.0): 6 (14.0)
Length of stay (days)	7.9 ± 4.0 (5-29) (Median: 7.0)
ICU/HDU stay (days)	3.3 ± 4.2 (0-26) (Median: 2.0)

Twenty-six (60.5%) patients had an uneventful postoperative course. Seven (16.3%) patients experienced minor deviations from the routine postoperative course and were classified as grade 1 in the Clavien-Dindo classification (Table 3). These included radiological investigations such as fluoroscopy or CT scan and cardiac enzymes, which were found to be negative. Higher Clavien-Dindo grades were found in 12 (27.9%) cases, indicating greater morbidity. There were no cases of anastomotic leak or chyle leak in this study.

**Table 3:** Complications. N, number of cases

Type of complication	*N* (%)
Clavien-Dindo classification	
Grade 1	7 (16.3)
Grade 2	3 (7.0)
Grade 3a	3 (7.0)
Grade 3b	1 (2.3)
Grade 4a	3 (7.0)
Grade 4b	1 (2.3)
Grade 5	1 (2.3)
Specific complications (isolated or in combination)	
Pleural effusion (requiring thoracostomy)	4 (9.3)
Pneumonia	1 (2.3)
Prolonged intubation	2 (4.7)
Prolonged intubation (requiring tracheostomy)	2 (4.7)
Delayed gastric emptying	2 (4.7)
Upper GI Endoscopy	3 (7.0)
Dysrhythmia	2 (4.7)
Myocardial Infarction	2 (4.7)
Acute kidney injury	1 (2.3)
Readmission	4 (9.3)
Re-exploration	1 (2.3)
Death	1 (2.3)

One patient had a non-ST-elevation myocardial infarction (NSTEMI) in the immediate postoperative period. This was managed medically; however, the patient subsequently developed acute tubular necrosis requiring hemodialysis. The same patient was later readmitted with fluid overload and required further hemodialysis. Another patient developed delayed gastric emptying, which necessitated multiple endoscopic interventions. One patient developed delayed gastric emptying; endoscopy revealed a patent lumen, which was nevertheless dilated. However, symptoms persisted, and fluoroscopy was suggestive of a stricture. So, the patient underwent surgical intervention two weeks after esophagectomy. Laparotomy revealed a constriction at the level of the diaphragm, but an unremarkable gastric conduit. Gastropexy was done, and a feeding jejunostomy was made, with resolution of the symptoms.

There was one mortality. The patient had suffered from COVID-19 pneumonia one month before surgery. Preoperatively, reassessment was done, and the patient was found to have recovered clinically, with resolution of pulmonary effects of COVID-19 on high-resolution computed tomography (HRCT) of the chest, although there were some radiation-induced changes in the lungs. However, the patient developed respiratory failure in the early postoperative course and died on the sixth postoperative day.

Table 4 demonstrates the relationship between complication rates and baseline factors. No gender predisposition was found in the incidence of complications. There was no association between the presence or absence of comorbidities and the development of complications. Similarly, complications were not associated with the underlying pathology, with similar rates in SCC as in AC.

**Table 4:** Factors affecting complications. N, number of cases; SCC, squamous cell carcinoma; AC, adenocarcinoma

Parameter	Complication occurred	No complication	Chi-square test	*P*-value
Male *N* (%): Female *N* (%)	12 (27.9): 7 (16.3)	19 (44.2): 5 (11.6)	χ2(1, *N* = 43) = 1.351	0.245
Comorbidities *N* (%): No comorbidities *N* (%)	13 (30.2): 6 (14.0)	12 (27.9): 12 (27.9)	χ2(1, *N* = 43) = 1.479	0.224
SCC *N* (%): AC *N* (%)	8 (18.6): 11 (25.6)	8 (18.6): 16 (37.2)	χ2(1, *N* = 43) = 0.349	0.555

Final histopathological outcomes are shown in Table 5. A complete response to neoadjuvant therapy was observed in six (14.0%) cases, with a near-complete response in one (2.3%) case. The majority of cases were T3 tumors, accounting for 21 (48.8%), while the most common grade was grade 2, with 25 (58.1%) cases. Lymph node involvement was absent in 17 (39.5%) patients. The most common final stage was IIIb, found in 19 (44.2%) cases.

**Table 5:** Pathology. N, number of cases; M, mean; SD, standard deviation; AJCC, American Joint Committee on Cancer staging manual for esophageal cancer (8th edition) [12]

Variable	*N* (%)/*M* ± SD
Histopathological type	
Adenocarcinoma	27 (62.8)
Squamous cell carcinoma	16 (37.2)
Lymph node yield	15.7 ± 7.7
Completeness of resection	
R0	36 (83.7)
R1	6 (14.0)
R2	1 (2.3)
Histological grade	
Complete/Near-complete response	7 (16.3)
Grade 1	2 (4.7)
Grade 2	25 (58.1)
Grade 3	9 (20.9)
Final T-stage (AJCC 8th Edition)	
0	6 (14.0)
1	3 (7.0)
2	11 (25.6)
3	21 (48.8)
4	2 (4.7)
Final N-stage (AJCC 8th Edition)	
0	17 (39.5)
1	7 (16.3)
2	15 (34.9)
3	4 (9.3)
Final TNM-stage (AJCC 8th Edition)	
I	12 (27.9)
II	6 (14.0)
IIIa	1 (2.3)
IIIb	19 (44.2)
IVa	5 (11.6)

R0 resection was achieved in 36 (83.7%) cases, and R1 in six (14.0%). There was a single case of R2 resection. This patient had been managed with definitive CRT at another hospital, but had then developed a recurrent mass. Salvage esophagectomy was done, but due to the severe fibrosis and locally advanced nature of the disease, complete resection was not possible.

## Discussion

Esophagectomy, along with CRT, forms the mainstay of management for EC [4]. It can be performed using several different techniques [14-16]. Several factors influence the type of surgery performed in any given case, including the tumor location, surgeon preference, and the availability of necessary expertise and equipment. However, regardless of the technique used, there is considerable morbidity and mortality associated with esophagectomy. Minimally invasive techniques involving laparoscopy, thoracoscopy, and robotic surgery have resulted in decreased levels of morbidity while achieving similar oncological outcomes, such as lymph node yield and survival rates [16,17]. These include entirely minimally invasive procedures as well as hybrid procedures, such as ILE performed at our center, which combines laparoscopy and thoracotomy.

ILE was the most common procedure done for EC at our center, accounting for almost 80% of all cases. The mean age of patients was 62 years. This is similar to global trends, with other studies reporting a mean age between 51 and 65 [9,14,15,17-20]. EC was more common in males, accounting for 72.1% of patients in our study. There were 31 (72.1%) male patients. There is some variation in the gender distribution of EC in different studies. While most researchers have found a higher incidence of the disease among men [14,15,17,19-21], some have reported that the converse is true [9].

AC (27, 62.8%) was almost twice as common as SCC (16, 37.2%) in our study. While SCC is more common than AC worldwide, comprising 85% of all cases of EC in 2019, there is considerable geographical variation in the ratio of SCC to AC, with a rising incidence of AC and a falling incidence of SCC in developed countries [1]. Different proportions of SCC and AC have been reported by researchers, with some finding a higher incidence of AC [15,19], while others have found a higher incidence of SCC [9,14,18,21].

The mean number of lymph nodes harvested was 15.7 ± 7.7. This is above the minimum yield of 15 lymph nodes as recommended by most guidelines [22,23]. Recently, some are advocating for the minimum number of lymph nodes to be resected in esophagectomy to be higher than this. Uimonen et al. suggest a minimum of 24 lymph nodes to be resected, which can result in improved survival, regardless of the nodes being involved by metastasis [24]. Higher lymph node yields should certainly be targeted, as low lymph node yields are associated with reduced overall survival [23,24]. We had a single case of R2 resection, where salvage esophagectomy was done for recurrence following definitive CRT. The locally advanced nature of the disease, with adherence to the aorta, coupled with fibrosis, limited safe dissection. Morita et al. studied definitive CRT for EC and reported R1/R2 resection in 29.6% of patients who underwent salvage esophagectomy after definitive CRT [18]. They found that the main reason for this was invasion of the surrounding viscera, mainly the aorta (as in our case), as well as the trachea or main bronchus.

Routine pyloric drainage procedures were not performed at our center. There is some controversy regarding its role in esophagectomy. While proponents suggest that it can improve gastric emptying, others claim that it may do more harm than good. Palmes et al. studied the effect of pyloric drainage in the form of pyloroplasty and pyloromyotomy in 198 patients undergoing esophagectomy [25]. They found that patients who underwent pyloric drainage had higher rates of bile reflux (14.9%, *P* = 0.069) and reflux esophagitis (34.5%, *P* < 0.05) compared to those who underwent esophagectomy without pyloric drainage procedures. Furthermore, they found no difference in the rates of gastrointestinal passage, anastomotic leak, hospital mortality, and the length of postoperative hospital stay among the groups.

There were two (4.7%) cases of delayed gastric emptying in our center. While one of these required surgical intervention in the form of gastropexy, the other was managed endoscopically. The latter underwent three endoscopic interventions; first, a balloon dilatation on the eighth postoperative day, followed by endoscopic balloon dilatation with botulinum toxin injection two weeks after the surgery. However, due to persistent symptoms, endoscopic placement of a fully covered duodenal stent (nitinol) was performed, which finally resulted in the resolution of symptoms. Bhutani et al. reviewed 21 patients who underwent endoscopic balloon dilatation along with botulinum toxin injections following esophagectomy, with improvement in 85% of patients. However, while 43% of patients underwent only one procedure, another 43% had two procedures performed, and 14% required multiple procedures [26].

Major complications (Clavien-Dindo grade 2 or higher) occurred in 12 (27.9%) cases in this study. These included mainly respiratory issues such as pleural effusions requiring surgical or interventional radiology (IR) guided chest drainage (4, 9.3%), prolonged intubation (4, 9.3%), and pneumonia (1, 2.3%). There were also cases of delayed gastric emptying (2, 4.7%), cardiac issues such as myocardial infarction (2, 4.7%), one reintervention (1, 2.3%), and one mortality (1, 2.3%). Other studies have reported similar rates of morbidity. Rizvi et al. reported morbidity in 31.37% of cases [9]; Bjelović et al. reported an overall morbidity of 27% [20]; Wang et al. reported a morbidity rate of 27.5% [17]; and Zheng et al. observed major surgical complications in 36.2% of cases [14]. Certain complications associated with esophagectomy, such as anastomotic leak and chyle leak, were not encountered in this study. However, the reason for this is not entirely clear. Shafiq et al. experienced a higher rate of anastomotic leaks (30, 5.1%); however, they did not identify any specific predisposing factors during either the surgery or the postoperative course [10]. There was one case (2.3%) in which reintervention was required. Rates of reintervention vary widely in different studies, and are most commonly due to hemorrhage [9,17]. In our study, the only patient who required a reintervention did so for delayed gastric emptying that had failed to respond to conservative management.

There are very few studies assessing esophagectomy outcomes in Pakistan. This study aims to bridge the gap in the literature and potentially lead to more regional research on this aspect of the disease. However, there are some significant weaknesses in this study. One major weakness of the study is its small sample size (from a single center), which limited the application of analytical statistics in the results. An inherent weakness of the study is its retrospective nature. Another potential weakness of the study is the absence of long-term outcomes and complications, as we focused more on the short-term postoperative course.

## Conclusions

This study highlights the feasibility of esophagectomy, including both conventional and minimally invasive approaches, in a developing country like Pakistan. Reasonable rates of morbidity and sound short-term oncological outcomes were achieved. Complications were found to be independent of gender, comorbidities, and the underlying pathology. Furthermore, when complications arose, they were mostly managed using conservative measures such as endoscopy and IR-guided drainage. However, the study is considerably constrained by the small sample size and relatively short follow-up. Therefore, larger, prospective, and ideally multicenter studies with a longer follow-up period are needed from this region.
